# Bergbaukunde, quo vadis?

**DOI:** 10.1007/s00501-023-01322-x

**Published:** 2023-01-27

**Authors:** Michael Tost, Philipp Hartlieb, Christian Heiss, Birgit Knoll, Tobias Ladinig, Gerhard Mayer, Peter Moser, Michael Prenner, Nikolaus Sifferlinger, Alexander Tscharf

**Affiliations:** Lehrstuhl für Bergbaukunde, Bergtechnik und Bergwirtschaft, Montanuniversität Leoben, Franz Josef Straße 18, 8700 Leoben, Österreich

**Keywords:** Chair of Mining Engineering and Mineral Economics, Strategy, Research, Education, Bergbaukunde, Strategie, Forschung, Lehre

## Abstract

Um dem Klimawandel und dem zunehmenden Wettbewerb beim Zugang zu Ressourcen zu begegnen, hat die Europäische Union im sogenannten Green Deal weitreichende Transformationen in den Bereichen Energie und Digitalisierung sowie die Umstellung des Wirtschaftssystems hin zu einer inklusiven Kreislaufwirtschaft festgelegt. Aus Sicht des Bergbaus bedeuten diese Transformationen einen weiterhin wachsenden Rohstoffbedarf, der durch die Gewinnung mineralischer Rohstoffe aus primären Lagerstätten gedeckt werden muss. Gesteigerte Mengen mit „Business as usual“ würden allerdings auch die Umwelt- und Sozialauswirkungen des Bergbaus steigern, was vor allem in Europa und Österreich keine Option darstellt.

Aufgrund dieser Tatsachen ergibt sich Forschungsbedarf für neue und verbesserte Abbauverfahren und -planung ebenso wie für optimierte Prozesse und Maschinen. Im vorliegenden Artikel wird beschrieben, wie der Lehrstuhl für Bergbaukunde, Bergtechnik und Bergwirtschaft an der Montanuniversität Leoben mit seiner aktuellen Strategie, sowohl für die Forschung als auch für die Lehre, diese Herausforderungen als Chance nutzen möchte. Für den Forschungsbereich soll dabei die Nachhaltigkeit und Involvierung in die Gestaltung von Rohstoffpolitik den Rahmen bilden. Mit einer Fokussierung auf die Digitalisierung und den untertägigen Bergbau sowie der Stärkung der Bereiche Tagebau, Fördertechnik und Geoinformatik soll die Forschung für die anstehenden Transformationen zukunftssicher ausgerichtet werden. Die Lehre wird inhaltlich weiterhin eine ingenieurwissenschaftliche Grundausbildung mit Spezialisierung auf den Bergbau beinhalten, wobei die Digitalisierung eine zunehmend stärkere Rolle spielen wird.

## Abstract (en)

In order to tackle climate change and increasing competition in access to resources, the European Union has defined far-reaching transformations in the areas of energy and digitalisation as well as the conversion of the economic system towards an inclusive, circular economy in the so-called Green Deal. From a mining perspective, these transformations mean a continued growing demand for raw materials, which must be met by extracting mineral raw materials from primary deposits. However, increased quantities with “business as usual” would also increase the environmental and social impacts of mining, which is not an option, especially in Europe and Austria.

Due to these facts, there is a need for research on new and improved mining methods and planning, as well as on optimised processes and machinery. This article describes how the Chair of Mining Engineering and Mineral Economics at Montanuniversität Leoben intends to use these challenges as an opportunity with its updated strategy, both for research and for teaching. For the research area, sustainability and involvement in the shaping of raw materials policy should form the framework. With a focus on digitisation and underground mining as well as strengthening the areas of open-pit mining, conveying technology and geoinformatics, research is to be future-proofed for these upcoming transformations. Teaching will continue to include a basic engineering education with specialisation in mining, whereby digitalisation will play an increasingly strong role.

## Einleitung

Das Jahr 2022 wird in die Geschichte als außergewöhnliches Jahr eingehen. Die Auswirkungen der Covid 19 Pandemie sind immer noch spürbar, der Klimawandel macht sich immer stärker – sowohl in unserer Umwelt als auch in unserer Gesellschaft (Stichwort „Letzte Generation“) – bemerkbar, und zu allem Überfluss gibt es seit Februar 2022 Krieg in der Ukraine. Die Konsequenzen für Europa, und damit auch für Österreich, sind signifikant. Vor allem im Bereich der Energiebereitstellung wurde uns vor Augen geführt, wie abhängig wir von anderen Ländern sind. Dies betrifft sowohl fossile Energierohstoffe, wie Öl und Gas, als auch den Europäischen Green Deal [1] und die damit verbundene Energiewende hin zu erneuerbaren Energien, wo sowohl im Bereich der benötigten Rohstoffe (z. B. Lithium, Kupfer, Seltene Erden) als auch bei vielen Technologien (z. B. Photovoltaik (PV), Batterien) eine massive Abhängigkeit besteht. Zusätzlich wird sich in den nächsten Jahren die Konkurrenz betreffend der Rohstoffbeschaffung aufgrund einer steigenden globalen Nachfrage wohl weiter verstärken. Die Weltbank [2] geht in einer Studie davon aus, dass sich allein der Bedarf für 17 mineralische Rohstoffe (inkl. Stahl, ohne Baurohstoffe), die für die Energiewende und das Erreichen des 2 °C Zieles des Paris Abkommens kritisch sind, bis 2050 vervierfachen wird. Die Produktion von Graphit, Lithium und Kobalt muss dafür um über 450 % gesteigert werden. Für Aluminium und Kupfer sind zwar die prognostizierten Zuwachsraten geringer, hier sind aber die absoluten Produktionszahlen, mit 103 Mt (2020: 65 Mt) und 29 Mt (2020: 21 Mt) im Jahr 2050, signifikant. Die Europäische Union (EU) geht in einer Studie zum Bedarf an kritischen Rohstoffen für strategisch relevante Sektoren (Erneuerbare Energie, E‑Mobilität, Verteidigung und Raumfahrt) und Technologien (u. a. Batterien, PV, Roboter, Halbleiter) von ähnlich hohen Wachstumsraten aus, welche nur zum Teil durch sekundäre Rohstoffe erfüllt werden können [3].

Die EU ist sich dieser Problematik durchaus bewusst und setzt seit über einem Jahrzehnt Maßnahmen im Bereich der mineralischen Rohstoffe, wie beispielsweise der Europäischen Innovationspartnerschaft für Rohstoffe (EIP RM, [4]), der Liste der kritischen Rohstoffe [5] oder der Europäischen Rohstoffallianz (ERMA, [6]), um sowohl Abhängigkeit als auch Konkurrenz zu begegnen. Dies soll durch vermehrten Bergbau in Europa selbst, Diversifizierung und strategische Partnerschaften mit Ländern außerhalb der EU sowie der Implementierung einer Kreislaufwirtschaft und verstärktem Recycling erfolgen. Durch den russischen Angriffskrieg dürfte sich das politische Momentum jetzt noch weiter erhöhen – der in Ausarbeitung befindliche „Critical Raw Materials Act“ ist ein Zeichen dafür.

Die Gewinnung mineralischer Rohstoffe aus primären Lagerstätten wird damit auch in Zukunft erforderlich sein, und bei vielen Rohstoffen wird es zu einem steigenden Bedarf kommen. Die Kehrseite dieser Medaille jedoch ist, dass die Umwelt- und Sozialauswirkungen des Bergbaus ebenfalls steigen werden. Dies ist vor allem in Europa, bedingt beispielsweise durch Green Deal und Vorgaben zur Klimaneutralität, eine bevorstehende Ausweitung von Natura 2000 Gebieten, Wasserrahmenrichtlinie und großteils geringer sozialer Akzeptanz des Bergbaus, aber auch global, keine Option. So propagiert die Weltbank in der oben angesprochenen Studie zum Bedarf von mineralischen Rohstoffen für die Energiewende eine „Climate Smart Mining“ Initiative, durch welche die CO_2_ Emissionen der Rohstoffgewinnung mit Hilfe der Digitalisierung und neuen Technologien signifikant gesenkt werden sollen. Verantwortungsvoller bzw. nachhaltiger Bergbau ist vor allem auch in der EU ein Thema, um einerseits die Umweltauswirkungen zu minimieren, aber andererseits auch um die soziale Akzeptanz für eine Ausweitung der Bergbauaktivitäten in Europa zu erhöhen. Die EU Prinzipien für nachhaltige Rohstoffe [7] und Projekte, wie die in einer früheren Ausgabe beschriebenen Projekte SUMEX [8] und Resourcing [9] oder auch MIREU^1^, wo Richtlinien für die Erhöhung der sozialen Akzeptanz von Bergbau in Europa erarbeitet wurden, seien hier neben zahlreichen Bestrebungen in den europäischen Rohstoffbetrieben selbst beispielhaft erwähnt. Österreich ist als handelsorientiertes Land stark von diesen Entwicklungen betroffen und nimmt in der EU durchaus auch eine aktive Rolle in der Gestaltung und Umsetzung der o. a. Rahmenbedingungen und Projekte ein.

Aus diesen Anforderungen für einen nachhaltigen Bergbau ergibt sich weiterer Forschungsbedarf hinsichtlich notwendiger Technologien sowie Bergtechnik, auf den in diesem Artikel im Kontext des Lehrstuhls für Bergbaukunde, Bergtechnik und Bergwirtschaft (BBK) der Montanuniversität Leoben näher eingegangen wird.

Forschung ist die eine wesentliche Kernaufgabe einer Universität, Lehre und die Ausbildung von Studierenden – in unserem Fall RohstoffingenieurInnen – die andere. Auch hier kommt es im Moment zu größeren Veränderungen. Global ist die Anzahl der Rohstoffstudierenden wie bei allen technischen und naturwissenschaftlichen Fächern rückläufig. In den letzten Jahren waren einige Universitäten dadurch gezwungen, ihre Studienangebote in den Bereichen Rohstoffe/Bergbau zu reduzieren oder einzustellen. Verstärkt wird diese Entwicklung auch durch große Bergbaugesellschaften, wie z. B. Rio Tinto, die davon ausgehen, dass in absehbarer Zukunft IT-IngenieurInnen, die Bergbau mithilfe von Digitalisierung, großen Datenmengen, künstlicher Intelligenz (KI) und Robotern betreiben, RohstoffingenieurInnen ersetzen – Helm und Sicherheitsschuhe werden sozusagen durch Tablets ersetzt. Die Societät der Bergbaukunde (SOMP), ein globaler Zusammenschluss von BergbauprofessorInnen, teilt diese Ansicht nicht vollinhaltlich, speziell was kleinere, komplexe oder untertägige Bergbaue sowie spezifische Prozesse wie geologisches Verständnis, Gebirgsmechanik und Bergbauplanung betrifft. Die SOMP geht aber ebenfalls davon aus, dass sich die Ausbildung ändern muss und dass neben ingenieur- und bergbaulichen Grundsätzen auch digitale Aspekte, von Sensorik über Robotik bis zu KI, und der gesellschaftliche und Umweltkontext, in dem Bergbau stattfindet, verstärkt vermittelt werden müssen. Helm und Sicherheitsschuhe werden nicht ersetzt, sondern mit modernen Inhalten und Methoden ergänzt [11].

**Figure 1:**
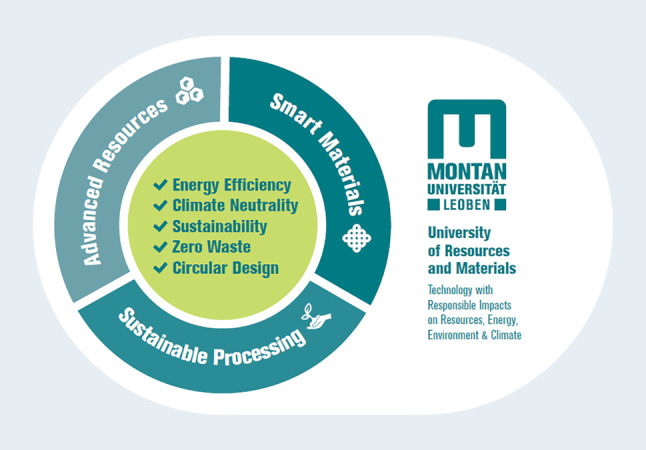

Im beschriebenen Kontext von Nachhaltigkeit und europäischem Green Deal, inklusive Klimaneutralität, Energiewende, Digitalisierung und Kreislaufwirtschaft, gesellschaftlichen Veränderungen und stagnierenden Studierendenzahlen hat die Montanuniversität Leoben Ende 2020 in ihrem Entwicklungsplan ihre Ziele und Strategien bis zum Jahr 2030 dargestellt [12]. Abb. 1 zeigt eine Zusammenfassung der strategischen Positionierung.

Für Bergbau ist im Kompetenzfeld „Advanced Resources“ folgender Ausrichtungsschwerpunkt beschrieben: *„Berg- und Tunnelbau mit besonderem Fokus auf neue ressourcen- und energieeffiziente Technologien, die Digitalisierung der Verfahrensprozesse, der Entwicklung neuer Abbauverfahren, die Geotechnik sowie die Ausrüstungs- und Sicherheitstechnik für Bau und Betrieb von Untertageanlagen.“* [12, S. 14]. Die Lehre soll inhaltlich auf diese Positionierung ausgerichtet sein, mit dem Ziel, 2030 die bevorzugte Bildungsinstitution für umweltbewusste Technikstudierende zu sein und die Anzahl der Studierenden auf 7000, 40 % davon international, zu erhöhen.

In diesem Artikel wird beschrieben, wie die BBK auf diese Rahmenbedingungen im Zuge einer aktualisierten strategischen Ausrichtung reagieren wird. Dazu wurde 2022 ein Strategieprozess durchlaufen; Abschn. 2 beschreibt die Methodik, Abschn. 3 das Resultat, und in Abschn. 4 werden die Ergebnisse im Kontext der beschriebenen Rahmenbedingungen beurteilt.

## Methodik

Der Strategieprozess wurde von einer internen Arbeitsgruppe der BBK, der alle wissenschaftlichen Mitglieder des Lehrstuhls mit Doktorat sowie die Studienverantwortliche angehörten, zwischen April und November 2022 durchgeführt. Betrachtet wurden dabei vornehmlich die zwei Kernaufgaben Forschung und Lehre. Mit externen Personen, im konkreten MitarbeiterInnen österreichischer und internationaler Bergbaufirmen, Interessensvertretungen, NGOs sowie MitarbeiterInnen der Montanbehörde wurden in diesem Zeitraum ergänzende Gespräche zu Teilbereichen der Strategie geführt bzw. wurde Feedback zu diversen Standpunkten eingeholt.

In einem ersten Schritt wurde eine SWOT (strengths/weaknesses/opportunities/threats) Analyse hinsichtlich der momentanen Aufstellung und Ausrichtung des Lehrstuhls aus interner Sicht (SW) und im oben beschriebenen externen Umfeld (OT) durchgeführt.

Danach wurde basierend auf den Ergebnissen der SWOT Analyse durch das AutorInnenteam über mehrere Monate in regelmäßigen Besprechungen eine sogenannte „Strategy on a page“ (SoaP) erstellt, welche die Vision, inhaltlichen Arbeitsbereiche und Schwerpunkte für Forschung und Lehre darlegt. Abschließend wurden für die jeweiligen Arbeitsbereiche detaillierte Schwerpunkte, mittel- (2028) und kurzfristige (2023) Ziele sowie die für die Umsetzung notwendigen zusätzlichen Ressourcen beschrieben.

## Vision – Ein global anerkannter Akteur in der Gestaltung des Bergbaus des 21. Jahrhunderts

**Figure 2:**
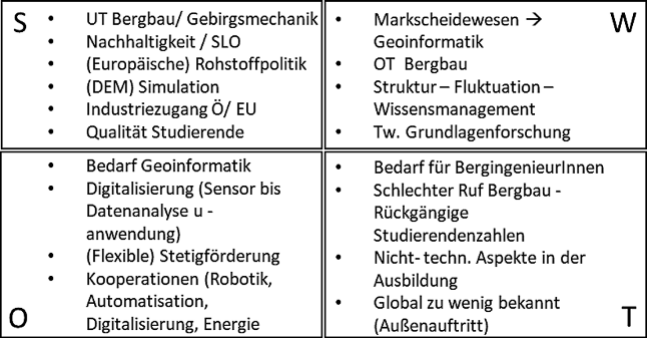

Das Ergebnis der SWOT Analyse ist in Abb. 2 zusammengefasst.

Basierend auf diesen Ergebnissen wurde die Vision für den Lehrstuhl für Bergbaukunde, Bergtechnik und Bergwirtschaft der Montanuniversität Leoben wie folgt festgelegt:

Ein global anerkannter Akteur in der Gestaltung des Bergbaus des 21. Jahrhunderts

**Figure 3:**
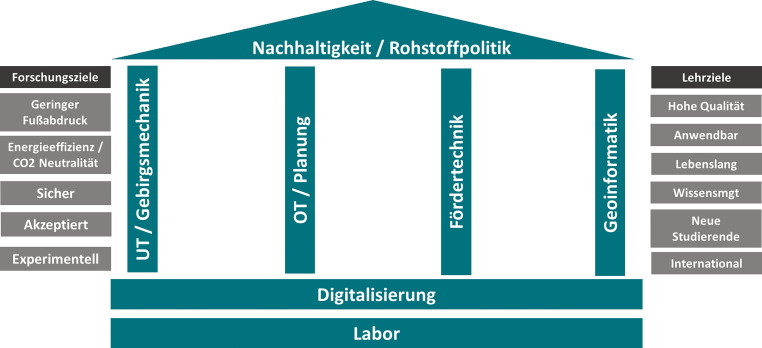

Die Umsetzung dieser Vision soll in sieben inhaltlichen Arbeitsbereichen erfolgen, welche in Abb. 3 in der Form eines Tempels dargestellt sind. Diese Arbeitsbereiche sind:

- Nachhaltigkeit/Rohstoffpolitik
- Untertagebergbau und Gebirgsmechanik
- Tagebau und Bergbauplanung
- Fördertechnik
- Geomatik und Geoinformatik
- Digitalisierung
- Labor

Nachhaltigkeit und Rohstoffpolitik bilden das übergeordnete Dach, Untertagebergbau/Gebirgsmechanik, Tagebau/Bergbauplanung, Fördertechnik sowie Geomatik und Geoinformatik sind die tragenden Säulen, welche auf zwei durchgängigen Fundamenten, dem Labor und der Digitalisierung, stehen. Als übergeordnete Forschungsziele für zukünftigen Bergbau wurden geringerer Fußabdruck (in Umweltbereichen wie Land- oder Wasserverbrauch, Bergbauabfälle, Luftemissionen, Auswirkungen auf Biodiversität), Energieeffizienz und CO_2_ Neutralität, Sicherheit, soziale Akzeptanz sowie experimentell (Grundlagenforschung für Verfahren, Prozesse, Bergbau in „remote areas“, etc) festgelegt. Übergeordnete Ziele für die Lehre sind eine hohe Qualität, die Anwendbarkeit der Ausbildung in der Praxis, v. a. in der Industrie und Verwaltung, die Schaffung von relevanten Inhalten und Formaten für lebenslanges Lernen, ein adäquates Wissensmanagementsystem, sowie ein Fokus auf die Gewinnung von neuen – und weiterhin auch internationalen – Studierenden. Um die Umsetzung dieser Ziele für den Bereich Lehre zu erreichen, wurde beschlossen, ebenfalls eine detaillierte Betrachtung durchzuführen.

Im Folgenden werden die Schwerpunkte und Ziele der einzelnen Bereiche zusammenfassend beschrieben. Diese ergaben sich aus der SWOT Analyse und basieren auf dem Prinzip, dass Stärken gestärkt und Schwächen minimiert werden sollen.

### Nachhaltigkeit und Rohstoffpolitik

Ziel ist es, eine aktive und europaweit anerkannte Rolle im rohstoffpolitischen Diskurs im Kontext Bergbau bzw. mineralische Rohstoffe und Nachhaltigkeit einzunehmen bzw. in weiterer Folge die Umsetzung von sich aus dem Diskurs ergebenden Zielsetzungen (z. B. CO_2_ Neutralität) zu begleiten. Schwerpunkte werden dabei die Rolle von mineralischen Rohstoffen im Kontext einer starken Nachhaltigkeit bzw. von Planetaren Grenzen (siehe SUMEX [8]), die Beurteilung von umwelt- und sozial-ökonomischen Auswirkungen von Bergbau (z. B. [13]), die soziale Akzeptanz (SLO) (siehe MIREU SLO Guidelines [10]), europäische und nationale Rohstoffpolitik (wie z. B. der „Critical Raw Materials Act“) sowie die notwendigen Rahmenbedingen für die Extraktion bzw. verantwortungsvolle Beschaffung von mineralischen Rohstoffen für die Energietransformation sein. Um dies zu ermöglichen, soll zu jedem Zeitpunkt mit einem Team von drei bis vier Personen weiterhin an zwei bis drei EU Projekten aktiv mitgearbeitet werden, und es sollen zusammen mit anderen Lehrstühlen der Montanuniversität Werkzeuge und Unterstützung für die Industrie entwickelt werden, wie beispielsweise für Lebenszyklusanalysen und Produktdeklarationen oder Nachhaltigkeits- und Energiepotentialanalysen.

### Untertagebergbau und Gebirgsmechanik

Der untertägige Abbau komplexer, tiefer oder auch durch Landnutzung eingeschränkter mineralischer Lagerstätten wird in Zukunft in Europa aufgrund der in der Einleitung dieses Artikels beschriebenen Rahmenbedingungen an Bedeutung gewinnen. Darum ist es das Ziel, diesen Bereich auszubauen und als „Untertagebergbau und Gebirgsmechanisches Zentrum“ in Europa zu etablieren. Dieses Zentrum soll eine enge Verknüpfung zur nationalen und internationalen Industrie aufweisen. Schwerpunkte des Zentrums werden sein:

- Forschung und Entwicklung betreffend Abbauverfahren, wie „raise caving“ [14], die sichererer, umweltverträglicher und kostengünstiger sein sollen
- Forschung und Entwicklung, um für die Industrie anwendbare, praktische Lösungen für ausgewählte Bereiche des untertägigen Bergbaus bereitzustellen, wie zum Beispiel standardisierte Planungskriterien und Planungsmethoden [15, 16], Verhalten von Gebirge, Verhalten und Auslegung von Festensystemen [17, 18], Abbaustrategien für die Bedingungen des tiefen Bergbaus [19], zielgerichtete Monitoringmaßnahmen und -programme sowie Auswertung von Monitoringdaten in Bezug auf die vorliegenden Gebirgsverhältnisse und die Abbauaktivität
- Fortlaufende Unterstützung von Industriebetrieben bei deren gebirgsmechanischen Fragestellungen an Hand von angewandter Forschung und Entwicklung.

Die Möglichkeiten der Digitalisierung und Automatisierung sollen dafür verstärkt genutzt werden. Ein Beispiel dafür sind digitale Gebirgsanker, welche derzeit entwickelt werden [20, 21]. In den letzten Jahren wurden mehrere Projekte bearbeitet, und es konnte ein Team bestehend aus sechs bis acht Positionen (Vollzeitäquivalent) aufgebaut werden. Das Team in diesem Bereich wird dazu in den nächsten Jahren massiv aufgestockt werden; es werden fünf bis sieben neue Positionen, von DissertantInnen bis zu Senior Positionen geschaffen sowie Synergien mit anderen Arbeitsbereichen innerhalb des Lehrstuhls genutzt und Partnerschaften mit anderen Lehrstühlen der Montanuniversität eingegangen. Längerfristig soll die Expertise in diesem Bereich auch dazu beitragen, den Lehrstuhl international, d. h. über Europa hinaus, bekannt zu machen.

### Tagebau und Bergbauplanung

Im Bereich Tagebau und Bergbauplanung sollen personelle Lücken, die in den vergangenen Jahren entstanden sind, durch organisatorische Änderungen und zusätzliche DissertantInnenstellen rasch wieder gefüllt werden.

Aktivitäten im Sektor Tagebau und Bergbauplanung werden zukünftig auf die Fest- und Lockergesteins- sowie der Werksteingewinnung in Österreich fokussiert sein, wobei Sicherheit und Risikomanagement im Bergbau, die Auswirkungen des Klimawandels auf Tagbaubetriebe und vice versa, Prozessoptimierungen hinsichtlich Nachhaltigkeit und Digitalisierung, sowie Bergbauschließung und -nachnutzung als konkrete Forschungsschwerpunkte festgelegt werden.

Die in den letzten Dekaden entwickelten Expertisen in den Bereichen Sprengtechnik, Bergbauplanung und Bergbausicherheit werden durch die Kooperation mit nationalen und internationalen Partner kontinuierlich erweitert und im universitären Regelbetrieb, aber auch in postgraduellen Lehrgängen, an zukünftige Generationen von Rohstoffingenieuren weitergegeben. So können z. B. vom Lehrstuhl entwickelte Prozesse und Verfahren – wie etwa das Böschungsbeurteilungsverfahren-Tagebau (B^2^ST) – rasch und unbürokratisch in der Praxis erprobt werden und Schulungen – wie z. B. der Universitätslehrgang Sprengtechnik – auch zünftig durch den Lehrstuhl für Bergbaukunde, Bergtechnik und Bergwirtschaft angeboten werden.

### Fördertechnik

Die Arbeitsgruppe Fördertechnik und Konstruktionslehre ist verantwortlich für die Lehre und Forschung

- der maschinen- und sicherheitstechnischen technischen Belange in der Rohstoffgewinnung
- im Montanmaschinenbau für den Studienzweig Schwermaschinenbau mit Schwerpunkten auf die Fördertechnik, mobile Montanmaschinen und Erdöl- und Erdgasförderung und zugehörige Sicherheitstechnik
- in der Industrielogistik für die Fördertechnik.

Die Schwerpunkte liegen im Bereich innovativer und nachhaltiger Lösungen, inkl. allen Aspekten der Simulation und Digitalisierung, von Sensorik und Monitoring (z. B. Zustandsüberwachung für Förderbänder) bis zur Anwendung von KI, Automatisierung (z. B. Automatisiertes Gurtspleißverfahren) und der Anwendung von Robotik im Bergbau [22–24]. Kontinuierliche und flexible Förderlösungen sollen zu kostengünstigen Alternativen zu SLKWs werden. Weitere Forschungsschwerpunkte sind Energieeffizienz und -rückgewinnung (z. B. mittels Feststoffturbinen) sowie die Reduktion von Staub, Lärm und Verschleiß. Zur Umsetzung der Ziele wird 2023 eine zusätzliche, global finanzierte DissertantInnenstelle eingerichtet.

### Geomatik und Geoinformatik

Die Erfüllung der gesetzlichen Anforderungen bezüglich verantwortlicher Personen im Markscheidewesen bildete – vor allem in der Lehre – den Fokus der letzten Jahre in diesem Bereich [25, 26]. Dies soll natürlich auch weiterhin gewährleistet sein und wird vor allem durch die Etablierung des internationalen Studienschwerpunkts „Geomatics for Mineral Resources Management“ in Kooperation mit den Universitäten in Freiberg, Wroclaw und Lissabon noch zusätzlich gestärkt und auf ein neues und modernes Niveau gehoben. Allerdings ergeben sich in diesem Bereich aufgrund der fortschreitenden Digitalisierung und der stetigen Neu- und Fortentwicklung von verschiedensten Sensoren auch zahlreiche Chancen, v. a. was die Zusammenarbeit mit Industrie, aber auch EU Projekte im Horizon Europe Programm betrifft. So soll beispielsweise die Nutzung von Erdbeobachtungsdaten im Bergbau künftig verstärkt in den Fokus rücken und somit die Forschungsarbeiten im Bereich der Nahbereichsfernerkundung und der Photogrammetrie [27, 28] fortgeführt werden. Schwerpunkte sind weiterhin

- die markscheiderische Sicherheits- und Betriebskontrolle (sowohl Ober- und Untertage)
- die Erstellung, Führung und Interpretation des Bergbaukartenwerks bzw. auch die Entwicklung und Nutzung digitaler Alternativen und Ergänzungen,
- die Prozesskontrolle und Betriebsdatenerfassung,
- die Ingenieurvermessung im Berg- und Tunnelbau und insbesondere die Veränderungs- und Deformationsvermessung,
- die Bergschadenkunde bzw. die Vorausberechnung von Bodenbewegungen und die Sicherung der Oberflächennutzung während und nach der Bergbautätigkeit sowie
- die Anwendung moderner Vermessungssysteme, sowohl im Nahbereich (Photogrammetrie, LiDAR) als auch Fernerkundung, GNSS und Hydrographie.

Damit wird die Tradition der Anwendung und Nutzung moderner geodätischer Verfahren zur Informationsbereitstellung und Interpretation im Bergbau fortgesetzt und an die Forschungsarbeiten der letzten Jahre angeknüpft bzw. der Aktionsradius erweitert und neue Potenziale erschlossen.

Zur Erreichung der angesprochenen Ziele und zur Gewährleistung der Qualität in Forschung und Lehre wird im Jahr 2023 in diesem Bereich eine zusätzliche Person auf Post-doc Level eingestellt, und überdies soll auch die vorhandene Infrastruktur aktualisiert bzw. mit moderner Vermessungsinfrastruktur (v. a. LiDAR) ergänzt werden.

### Digitalisierung

Selbstverständlich spielt Digitalisierung in den oben beschriebenen „Säulen“ des Lehrstuhls für Bergbaukunde, Bergtechnik und Bergwirtschaft bereits seit vielen Jahren eine signifikante Rolle, allerdings soll diese nun als eigener – alle „Säulen“ unterstützender – Arbeitsbereich weiter gestärkt werden. Keiner der oben genannten Schwerpunkte kommt ohne entsprechende digitale Kompetenzen aus. Dies betrifft zum einen die Entwicklung neuer Sensoren und deren Implementierung (Stichwort digitale Anker), zum andern aber auch die Zusammenführung der enormen Datenmengen, um eine umfassende Evaluierung zu ermöglichen. Schwerpunkte sind die Sensorik und Datenerfassung ausgewählter Prozesse (z. B. Gebirgsstabilität (Anker [21], B^2^ST) oder bildgebende Verfahren (Drohnen, Satelliten, andere Kamerasysteme [29, 30]), die Simulation (z. B. Gebirgsmechanik [31], Materialflüsse [32]), Datenmanagement und -analyse sowie die Integration der verschiedenen Datenquellen in Richtung „digital twin“ und Anwendung von „machine learning“ und künstlicher Intelligenz (KI) zur Evaluierung und Verbesserung von Prozessen, Bergbauplanung und -design [33, 34]. Auch in diesem Bereich sind Partnerschaften mit anderen Lehrstühlen der Montanuniversität, v. a. dem neuen Lehrstuhl für Cyber-Physical Systems und anderen Universitäten geplant. Zurzeit arbeitet ein Team von fünf bis sechs Personen direkt an diesem Querschnittsthema und ist hauptsächlich in Projekten (illuMINEation^2^, DigiEcoQuarry^3^, S34I^4^) auf Europäischem Level mit der Weiterentwicklung beschäftigt. Die entsprechende Infrastruktur soll laufend erweitert werden und der Personalstand soll gehalten werden.

### Labor

Das Labor sieht sich auch zukünftig primär als unterstützende Einheit lehrstuhlinterner und lehrstuhlexterner wissenschaftlicher MitarbeiterInnen der Montanuniversität bei ihrer Durchführung von Grundlagenforschung und angewandter Forschung. Durch eine institutsübergreifende Zusammenarbeit kann so – basierend auf interdisziplinärem Austausch und Synergien – wesentliches Know How in der Versuchsdurchführung, -dokumentation und -analyse geschaffen werden. Die Laboraktivitäten werden sich auch künftig an der bergmännischen Grundlagenforschung orientieren, wobei die Datenerfassung bei tatsächlichen Ereignissen, Prozessen oder Situationen in vielen Bereichen durch 1:1 Feldversuche und Versuche im Labormaßstab ergänzt werden, um so möglichst vertrauenswürdige Daten für weiterführende kalkulatorische Analysen zu generieren. Die Materialprüfung wird auch weiterhin eine Kernkompetenz des Labors darstellen. Die Möglichkeiten der Materialprüfung von Fest- und Lockergestein sollen im Labor daher derart erweitert bzw. ergänzt werden, dass Gesteinsproben künftig umfassend geprüft werden können. D. h. auch die Möglichkeit zur Durchführung von Scherversuchen sollte gegeben sein. Durch eine enge Zusammenarbeit mit Industriepartnern im aktuellen Forschungsprojet NAGEMA (Naturgefahren Management) hat der Lehrstuhl für Bergbaukunde ab 2024 auch die Möglichkeit, eine Versuchsanlage für dynamische Energieeinträge bis 10 MJ für die Entwicklung von Hangsicherungen und untertägige Ausbauvarianten zu nutzen. Mit dieser infrastrukturellen Erweiterung zur Durchführung von Versuchen im Maßstab 1:1 wird das Portfolio des Lehrstuhls wesentlich erweitert.

### Lehre

Wie bereits angeführt, sind die übergeordneten Ziele für die Lehre eine hohe Qualität der Inhalte, die Anwendbarkeit der Ausbildung in der Praxis, v. a. in der Industrie und Verwaltung, die Schaffung von relevanten Inhalten und Formaten für lebenslanges Lernen, ein adäquates Wissensmanagementsystem sowie der Fokus auf die Gewinnung von neuen – und weiterhin auch internationalen – Studierenden. Die hohe Qualität soll beispielsweise durch die Wiederbelebung der lehrstuhlinternen „Arbeitsgemeinschaft Lehre“, regelmäßige Meetings der Lehrenden und stärkere Einbeziehung der externen Lehrenden, zielgerichtete Nutzung der Evaluierungsergebnisse sowie einer transparenten Leistungsbeurteilung („Assessment Criteria“) erreicht werden. Inhaltlich soll weiterhin eine ingenieurwissenschaftliche Grundausbildung mit Spezialisierung auf den Bergbau und die o. a. angeführten Bereiche angeboten werden, wobei die Digitalisierung auch in der Ausbildung eine zunehmende Rolle spielen wird. Die Relevanz und Anwendbarkeit in der Praxis soll u. a. durch eine regelmäßige Evaluierung der Studien- und Lernziele unter Einbeziehung von VertreterInnen der Wirtschaft/Institutionen sowie der StudienrichtungsvertreterInnen geschehen. Neue Studienschwerpunkte des Masterstudiums werden mit dem „European Mining Course“^5^ und dem Ausbau der Geomatik gesetzt. Das Joint Master Degree Programm AMRD (Advanced Minerals Resources Development) wird mit der kenianischen Partneruniversität Taita Taveta University erweitert. In Übereinstimmung mit dem Entwicklungsplan der Montanuniversität wird unter dem Aspekt „life long learning“ 2023/24 evaluiert, ob es in Wirtschaft und Institutionen – zusätzlich zum bestehenden Universitätslehrgang Sprengtechnik – Bedarf für zusätzliche Weiterbildungsangebote gibt. Teile diverser Lehrinhalte des Lehrstuhls (z. B. Bergbau und Energie, Nachnutzung, Gebirgsmechanik, Nachhaltigkeit, Digitalisierung) könnten dann unter dem Titel einer Rohstoffakademie und in verschiedenen Formaten („Micro-creditentials“, online/„Massive Open Online Courses“, etc) angeboten werden. Ein Wissensmanagementsystem für den Lehrstuhl soll noch 2023 aufgebaut werden. Die Gewinnung neuer Studierender ist ein wesentliches Ziel der Montanuniversität. Unser Lehrstuhl wird dabei eine aktive Rolle einnehmen.

## Diskussion und Conclusio

Klimawandel, Ressourcenknappheit bzw. zunehmender Wettbewerb um den Zugang zu Ressourcen und geopolitische Überlegungen stellen für Europa und damit auch Österreich eine große Herausforderung dar, der mittels Green Deal [1] und den darin festgelegten Transformationen in den Bereichen Energie, Digitalisierung sowie des (linearen) Wirtschaftssystems hin zu einer inklusiven Kreislaufwirtschaft begegnet werden soll. Die Montanuniversität Leoben hat in Ihrem Entwicklungsplan [12] festgelegt, wie sie in den nächsten Jahren diesen Herausforderungen, inkl. der darauf beruhenden gesellschaftlichen Veränderungen, begegnen wird. Für die Universität, speziell aber für die Bergbaukunde, ergeben diese Transformationen auch eine Chance, da Studien davon ausgehen, dass der Bedarf für mineralische Rohstoffe, auch aus primären Quellen, weiterhin wachsen wird [2, 3]. Um diesen wachsenden Bedarf decken zu können, wird Bergbau sich allerdings wesentlich verändern (müssen), v. a. hinsichtlich Umweltauswirkungen und sozialer Akzeptanz. Neue und verbesserte, effizientere und sicherere Verfahren und Prozesse, digitalisiert und automatisiert, werden dabei eine wesentliche Rolle spielen, was zumindest den großen, globalen Bergbaufirmen durchaus bewusst ist^6^.

Durch die in diesem Artikel beschriebene Ausrichtung des Lehrstuhls für Bergbaukunde, Bergtechnik und Bergwirtschaft der Montanuniversität Leoben – mit Nachhaltigkeit als Rahmen, einer Fokussierung auf Digitalisierung und untertägigen Bergbau, Gebirgsmechanik sowie Stärkung der Bereiche Tagbau, Fördertechnik und Geoinformatik sollte die Forschung für die anstehenden Transformationen – und sich daraus ergebende notwendige Unterstützung bei der Lösung etwaiger Problemstellungen in der österreichischen und internationalen Rohstoffindustrie – zukunftssicher ausgerichtet sein. In der Lehre werden zum jetzigen Zeitpunkt Helm und Sicherheitsschuhe nicht ersetzt, sondern sukzessive mit dem Tablet ergänzt, was mit der Position von SOMP betreffend Ausbildung übereinstimmt [11]. Zusätzlich sollen in den nächsten Jahren weitere Ausbildungsangebote unter dem Aspekt „life long learning“ geschaffen und angeboten werden. Es ist den AutorInnen durchaus bewusst, dass mit dieser strategischen Ausrichtung nicht das gesamte Spektrum des Bergbaus abgedeckt wird (z. B. hinsichtlich deep sea mining); das Ziel ist aber, in den beschriebenen Bereichen eine tragende Rolle als global anerkannter Akteur in der Gestaltung des Bergbaus des 21. Jahrhunderts zu erreichen.

## Footnotes

1 https://mireu.eu/.

2 https://www.illumineation-h2020.eu/.

3 https://digiecoquarry.eu/.

4 Startdatum 01.01.2023 – noch keine Online Informationen verfügbar.

5 https://www.emc-master.eu/courses.

6 Siehe beispielsweise https://www.angloamerican.com/futuresmart/futuresmart-mining.
